# The Antioxidant, Anti-Inflammatory, and Neuroprotective Properties of the Synthetic Chalcone Derivative AN07

**DOI:** 10.3390/molecules25122907

**Published:** 2020-06-24

**Authors:** Yih-Fung Chen, Sheng-Nan Wu, Jia-Mao Gao, Zhi-Yao Liao, Yu-Ting Tseng, Ferenc Fülöp, Fang-Rong Chang, Yi-Ching Lo

**Affiliations:** 1 Graduate Institute of Natural Products, College of Pharmacy, Kaohsiung Medical University, Kaohsiung 80708, Taiwan; monica.ttgfc@gmail.com (Y.-F.C.); aaronfrc@kmu.edu.tw (F.-R.C.); 2 Department of Medical Research, Kaohsiung Medical University Hospital, Kaohsiung 80708, Taiwan; 3 Drug Development and Value Creation Research Center, Kaohsiung Medical University, Kaohsiung 80708, Taiwan; 4 Department of Physiology, National Cheng Kung University Medical College, Tainan City 70101, Taiwan; snwu@mail.ncku.edu.tw; 5 Department of Pharmacology, School of Medicine, College of Medicine, Kaohsiung Medical University, Kaohsiung 80708, Taiwan; kira771121@gmail.com (J.-M.G.); yeats1122@hotmail.com (Z.-Y.L.); mmship1112@yahoo.com.tw (Y.-T.T.); 6 Institute of Pharmaceutical Chemistry, University of Szeged, Eötvös u. 6, H-6720 Szeged, Hungary; fulop@pharm.u-szeged.hu; 7 MTA-SZTE Stereochemistry Research Group, Hungarian Academy of Sciences, Eötvös u. 6, H-6720 Szeged, Hungary; 8 Graduate Institute of Medicine, College of Medicine, Kaohsiung Medical University, Kaohsiung 80708, Taiwan

**Keywords:** chalcone, lipopolysaccharide, inflammation, oxidative stress, methylglyoxal, neuroprotection

## Abstract

Chalcones belong to a class of biologically active polyphenolic natural products. As a result of their simple chemical nature, they are easily synthesized and show a variety of promising biological activities. 2-Hydroxy-4′-methoxychalcone (AN07) is a synthetic chalcone derivate with potential anti-atherosclerosis effects. In this study, we demonstrated the novel antioxidant, anti-inflammatory, and neuroprotective effects of AN07. In RAW 264.7 macrophages, AN07 attenuated lipopolysaccharide (LPS)-induced elevations in reactive oxygen species (ROS) level and oxidative stress via down-regulating gp91^phox^ expression and stimulating the antioxidant system of nuclear factor erythroid 2-related factor 2 (Nrf2) and heme oxygenase-1 (HO-1) pathways, which were accompanied by increased glutathione (GSH) levels. Additionally, AN07 attenuated LPS-induced inflammatory factors, including NO, inducible NO synthase (iNOS), cyclooxygenase-2 (COX-2), and phosphorylated inhibitor of nuclear factor kappa B-alpha (p-IκBα) in RAW 264.7 macrophages. However, the effects of AN07 on promoting nuclear Nrf2 levels and decreasing COX-2 expressions were significantly abrogated by the peroxisome proliferator-activated receptor-γ (PPARγ) antagonist GW9662. In human dopaminergic SH-SY5Y cells treated with or without methylglyoxal (MG), a toxic endogenous by-product of glycolysis, AN07 up-regulated neurotrophic signals including insulin-like growth factor 1 receptor (IGF-1R), *p*-Akt, *p*-GSK3β, glucagon-like peptide 1 receptor (GLP-1R), and brain-derived neurotrophic factor (BDNF). AN07 attenuated MG-induced apoptosis by up-regulating the B-cell lymphoma 2 (Bcl-2) protein and down-regulating the cytosolic expression of cytochrome c. AN07 also attenuated MG-induced neurite damage via down-regulating the Rho-associated protein kinase 2 (ROCK2)/phosphorylated LIM kinase 1 (p-LIMK1) pathway. Moreover, AN07 ameliorated the MG-induced down-regulation of neuroprotective Parkinsonism-associated proteins *parkin*, *pink1*, and *DJ-1*. These findings suggest that AN07 possesses the potentials to be an anti-inflammatory, antioxidant, and neuroprotective agent

## 1. Introduction

**Figure 1:**
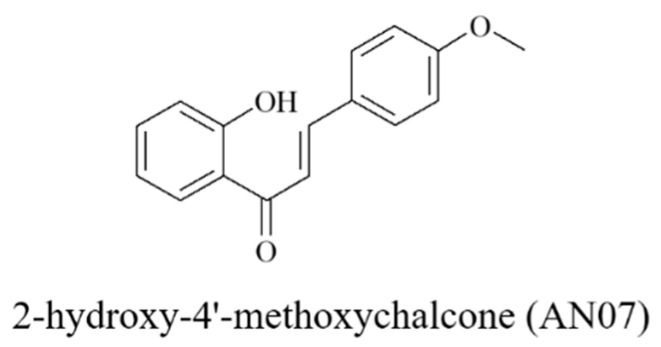
Chemical structure of 2-hydroxy-4′-methoxychalcone (AN07).

Chalcones (1,3-diphenyl-2-propen-1-ones) are a subclass of open-chain flavonoids that can be found in many naturally occurring compounds or manufactured synthetically [1]. Chalcones are regarded as a privileged scaffold in medicinal chemistry and widely used as an effective template for drug discovery [1]. As a result of their simple chemistry and convenient synthesis, many chalcone derivatives have been prepared and exert a broad spectrum of activities such as anti-inflammatory, antioxidant, anti-tumor, anti-microbial, and neuroprotective activities in different biological systems [2,3]. Some clinical trials have shown that compounds with the chalcone skeleton can reach sufficient plasma concentrations without causing significant toxicities [2,4,5]. Therefore, increasing interest in the potentials of chalcone derivatives has been gained from both academia and industry. Their promising biological profiles, along with their ease of synthetic manipulations, motivated us to develop a series of novel chalcone-based compounds with multifarious protective activities in single molecules [6,7]. Among these novel chalcone derivatives, 2-hydroxy-4′-methoxychalcone (AN07, Figure 1) has been demonstrated to possess the anti-inflammatory and anti-atherosclerotic activities of AN07 in human aortic smooth muscle cells via the stimulation of peroxisome proliferator-activated receptor gamma (PPARγ) expressions [7]. However, the anti-oxidative properties and neuronal effects of AN07 have not been explored.

Persistent or prolonged inflammation plays a prominent role in the development and progression of many diseases. The overproduction of pro-inflammatory mediators in uncontrolled inflammation leads to various pathological lesions, including cardiovascular, diabetic, and neurodegenerative complications. Monocyte-derived macrophages are important immune regulatory cells that play essential roles in inflammatory responses by producing various inflammatory mediators, such as nitric oxide (NO) and prostaglandin E_2_ (PGE_2_), as well as different pro-inflammatory cytokines, such as tumor necrosis factor-alpha (TNFα), interleukin (IL)-1β, and IL-6 [8]. Lipopolysaccharide (LPS), the main component of endotoxin from Gram-negative bacteria, is a well-known and potent trigger of inflammatory responses of macrophages [9]. Additionally, it is well recognized that inflammation is associated with oxidative stress. Oxidative stress refers to elevated intracellular levels of reactive oxygen species (ROS) that are considered to be the potent inflammatory mediators [10]. Nrf2 is known to regulate the transcription of proteins involved in maintaining cellular redox and counteracting oxidative damage in many types of cells, including macrophages [11]. Under basal conditions, Nrf2 is sequestered in the cytoplasm through the interaction with Keap1 (Kelch-associated protein 1) protein. When oxidative stimuli occur, Nrf2 translocates into the nucleus due to the dissociation of Nrf2 and Keap1, and then it further activates the expression of downstream proteins involving antioxidant defense systems. Antioxidant proteins activated by Nrf2 include heme oxygenase-1 (HO-1), which degrades toxic heme and generates the antioxidant molecules biliverdin and carbon monoxide (CO) [12], as well as glutathione (GSH)-generating enzymes [13]. GSH is the primary intracellular antioxidant that can directly scavenge ROS and thereby prevent oxidative stress. Increased GSH levels in the LPS-stimulated macrophages are found to alleviate oxidative stress-related inflammatory damages [14]. In this study, the protective effects of the chalcone derivative AN07 against inflammatory disorders and oxidative stress products were examined in the model of LPS-stimulated macrophages.

Accumulating studies have demonstrated that the formation of advanced glycation end products (AGEs) induced by hyperglycemia contributes to the development of many diabetes-related complications, including hyperglycemia-associated neuronal damage [15]. AGEs are generated from the reaction of the carbonyl groups of the reducing sugars and the free amino groups of the proteins, which modify protein half-life, alter enzyme activity, and change protein immunogenicity [16]. Methylglyoxal (MG), which is identified as a potent reactive dicarbonyl precursor of AGEs, is a metabolic by-product produced during glycolysis and causes serious toxicological effects when in excess [17]. Elevated levels of MG in the tissues or plasma of animals or the exogenous administration of MG to the cultured cells recapitulate pathophysiological changes observed in diabetic patients and ultimately leads to the pathology of diabetic complications [18]. Regarding the neurotoxic mechanisms, MG has been reported to interfere with signaling pathways involved in cellular metabolism, inflammation, oxidative stress, and apoptosis, resulting in the hippocampal neuronal toxicity [17,19,20]. In addition, MG possesses highly selective neurotoxicity in spinal motor neurons for the inefficiency of the glutathione (GSH) system [21], and it also contributes to limited neurite outgrowth and apoptotic death in cultured motor neuron-like cells [22]. Here, the protective effects of the chalcone derivative AN07 against MG-induced neuronal damage in human dopaminergic cells were examined.

The insulin-like growth factor-1 receptor (IGF-1R) and its downstream signaling cascades are not only essential for normal development of the central nervous system [23], but also important neuroprotective signaling in various neurodegenerative conditions [24]. Activation of the intrinsic tyrosine kinase activity of IGF-1R leads to the activation of phosphorylated 3-kinase (PI3K)/protein kinase B (Akt) and several downstream effectors related to protein synthesis, neurotrophic effects, neurite outgrowth, or anti-apoptotic effects [25,26]. In human dopaminergic SH-SY5Y cells, IGF-1 attenuated hyperglycemia-induced oxidative stress and neuronal injury via activation of the survival signaling cascade PI3K/Akt [27]. Previous studies suggested that drugs targeting IGF-1R-dependent signals possess the neuroprotective potential against diabetic-like neuronal apoptotic death and neurite damage induced by MG or high glucose [22,28]. Glucagon-like peptide 1 (GLP-1) is an endogenous insulinotropic peptide that is secreted by the intestinal endocrine cells with blood-glucose lowering effects by stimulating insulin secretion [29]. GLP-1 possesses similar functions and neurotrophic properties as IGF-1, and the dysfunction of either pathway leads to neurodegenerative disorders [30]. Studies in cellular and animal neurodegeneration models support a neurotrophic and neuroprotective effect of the GLP-1 receptor (GLP-1R) signaling and the potentials of GLP-1R agonists for the treatment of neurodegenerative disorders [31]. Brain-derived neurotrophic factor (BDNF) is a neurotrophic factor essential for neuronal development and survival [32,33]. Additionally, BDNF has been reported to be associated with the activation of Akt/GSK-3β, the downstream signals of IGF-1R and GLP-1R [34]. Activation of the BDNF and Akt/GSK-3β signaling pathway has been implicated in the drug-mediated neuroprotective effects, including neuronal survival, neurite outgrowth, and other neurotrophic effects [35].

The present study is the first to reveal the novel multifarious protective effects and the underlying mechanisms of the chalcone derivative AN07 against LPS-induced oxidative stress and inflammatory responses and MG-induced neurotoxicity. We demonstrated that AN07 exerts beneficial effects on anti-inflammatory reactions and antioxidant defense in LPS-induced RAW 264.7 macrophages. AN07 also protects human dopaminergic SH-SY5Y cells against MG-induced stress by mechanisms involving the up-regulation of cell survival, neurite outgrowth, and neurotrophic signals.

## 2. Results

### 2.1. AN07 Attenuates LPS-Induced Oxidative Stress and Enhances Antioxidant Defense in RAW 264.7 Macrophages

**Figure 2:**
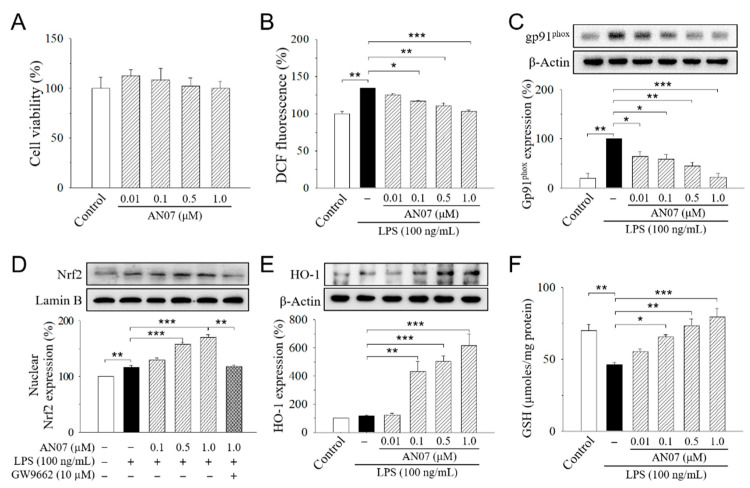
2-Hydroxy-4′-methoxychalcone (AN07) protects from lipopolysaccharide (LPS)-induced oxidative stress in RAW 264.7 cells. (**A**) Effects of AN07 on cell viability of RAW264.7 cells. RAW264.7 cells were treated with AN07 (0.01–1.0 μM) for 24 h, and cell viability was determined by 3-(4,5-dimethylthiazol-2-yl)-2,5-diphenyl-tetrazolium bromide (MTT) assay. (**B**–**F**) AN07 attenuates reactive oxygen species (ROS) production (**B**), reduces gp91^phox^ protein expression (**C**), enhances the nuclear levels of the nuclear erythroid 2-related factor 2 (Nrf2) (**D**), increases heme oxygenase-1 (HO-1) expression (**E**), and rescues glutathione (GSH) depletion (**F**) in LPS-stimulated RAW264.7 cells. RAW264.7 cells were pre-treated with AN07 (0.01–1.0 μM) for 1 h and then exposed to LPS (100 ng/mL) for another 24 h. (**B**) ROS production was determined by Hoechst 33342, 2′,7′-dichlorodihydrofluorescein diacetate (H2DCF-DA) staining, and fluorescence of the 2′,7′-dichlorofluorescein (DCF) product was analyzed by flow cytometer, which was represented as percentages of the control group. (**C**–**E**) Upper panels, representative immunoblots. Lower panels, densitometry analyses of the relative ratio of gp91^phox^ protein/β-actin protein, Nrf2/Lamin B protein, or HO-1 protein/β-actin protein, represented as percentages of control or LPS group. GW9662 (10 μM), a selective peroxisome proliferator-activated receptor-γ (PPARγ) antagonist. (**F**) GSH levels were determined using the commercial kit. The total amount of GSH was determined through a calibration curve and normalized to the protein concentration. Columns, mean ± S.E.M. from at least three independent experiments. * *p* < 0.05, ** *p* < 0.01, *** *p* < 0.001, compared with control or indicated groups.

We first examined the anti-inflammatory and antioxidant potentials of AN07 by using LPS-stimulated RAW 264.7 cells. RAW 264.7 cells were treated with or without 0.01–1.0 μM AN07 for 1 h before the stimulation of LPS (100 ng/mL) for 24 h. Results from 3-(4,5-dimethylthiazol-2-yl)-2,5-diphenyl-tetrazolium bromide (MTT) assay indicated that the exposure of RAW 264.7 cells to AN07 at 0.01–1.0 μM did not affect cell viability (Figure 2A). We next investigated the effects of AN07 pre-treatment on LPS-induced ROS generation and the protein expressions of gp91^phox^ (NOX2), which is a major isoform of the macrophage nicotinamide adenine dinucleotide phosphate (NADPH) oxidase that mediates LPS-induced ROS. LPS-induced ROS production was measured by using Hoechst 33342, 2′,7′-dichlorodihydrofluorescein diacetate (H2DCF-DA) staining and detecting the 2′,7′-dichlorofluorescein (DCF) fluorescence. Results indicated that AN07 at 0.01–1.0 μM attenuated LPS-induced ROS overproduction in a concentration-dependent manner (Figure 2B), which is accompanied by the down-regulation of LPS-induced gp91^phox^ overexpression (Figure 2C).

We also evaluated the effects of AN07 on nuclear factor erythroid 2-related factor 2 (Nrf2), which is a key transcription factor that regulates the expression of proteins involved in maintaining cellular redox and counteracting oxidative damages in macrophages. In LPS-stimulated RAW 264.7 cells, AN07 pre-treatment at 0.01–1.0 μM enhanced the nuclear expression of Nrf2 in a concentration-dependent manner (Figure 2D), which was accompanied by an increased level of the downstream antioxidant protein heme oxygenase-1 (HO-1) (Figure 2E). Furthermore, AN07 pre-treatment significantly increased the levels of non-enzymatic antioxidant GSH in LPS-stimulated RAW 264.7 cells (Figure 2F).

### 2.2. AN07 Attenuates LPS-Induced Inflammation in RAW 264.7 Macrophages

**Figure 3:**
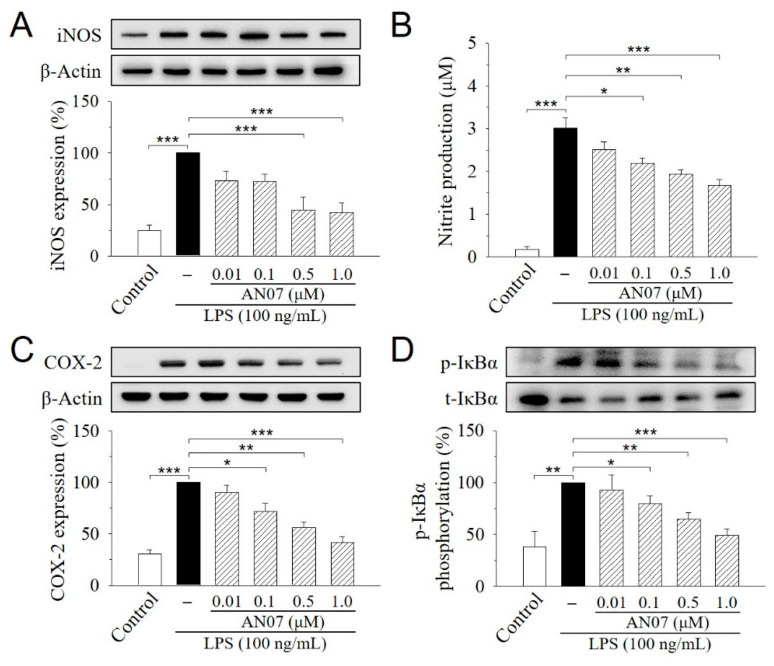
AN07 prevents lipopolysaccharide (LPS)-induced increase in the inducible nitric oxide synthase (iNOS) expression (**A**), NO production (**B**), cyclooxygenase-2 (COX2) expression (**C**), and inhibitor of nuclear factor kappa B-alpha (IκBα) phosphorylation (**D**) in RAW 264.7 cells. RAW 264.7 cells were pre-treated with AN07 (0.01–1.0 μM) for 1 h and then exposed to LPS (100 ng/mL) for another 24 h. (**A**,**C**,**D**) Upper panels, representative immunoblots. Lower panels, densitometry analyses of the relative ratio of protein/β-actin protein and phosphorylated-IκBα (p-IκBα)/total-IκBα (t-IκBα), which are represented as percentages of the LPS group. (**B**) Nitrite production was measured using the Griess reagent. The concentration was calculated from a standard curve of reference sodium nitrite. Columns, mean ± S.E.M. from at least three independent experiments. * *p* < 0.05, ** *p* < 0.01, *** *p* < 0.001.

We next studied the protective effects of AN07 on LPS-induced inflammatory responses in RAW 264.7 cells. During the inflammatory process, large amounts of the pro-inflammatory mediators are generated by the inducible nitric oxide synthase (iNOS) and cyclooxygenase-2 (COX-2). As shown in Figure 3A,C, iNOS and COX-2 proteins were increased in LPS-induced RAW 264.7 cells, while AN07 pre-treatment at 0.01–1.0 μM significantly decreased the elevated levels of iNOS and COX-2 proteins in a concentration-dependent manner. Additionally, the LPS-induced production of nitric oxide (NO), the downstream product of iNOS, was similarly decreased by AN07 pre-treatment (Figure 3B). Moreover, AN07 pre-treatment concentration-dependently attenuated LPS-induced inhibitor of nuclear factor kappa B-alpha (IκBα) phosphorylation in RAW 264.7 macrophages (Figure 3D), which has been linked to the suppression of a pro-inflammatory NF-κB signaling pathway. These results suggested that AN07 possesses antioxidant effects against LPS-induced inflammation and oxidative stress in RAW 264.7 macrophages.

**Figure 4:**
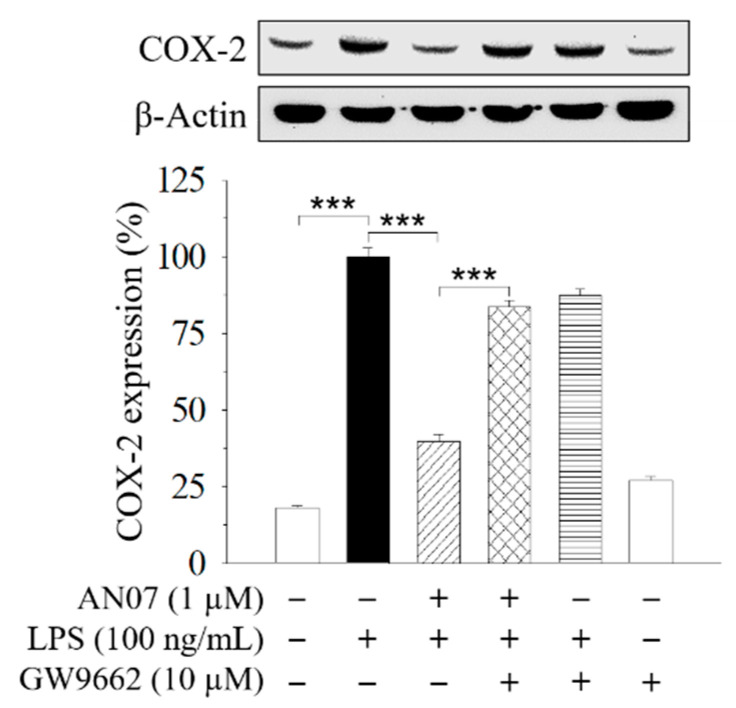
The inhibitory effects of AN07 on COX-2 expressions were attenuated by a selective PPARγ antagonist GW9662. RAW 264.7 cells were pre-treated with AN07 (1.0 μM) and/or GW9662 (10 μM) for 1 h and then exposed to LPS (100 ng/mL) for another 24 h. Upper panels, representative immunoblots. Lower panels, densitometry analyses of the relative ratio of protein/β-actin protein, represented as percentages of the LPS group. Columns, mean ± S.E.M. from at least three independent experiments. *** *p* < 0.001.

The previous study showed that the PPARγ-related pathway participates in the protective effect of AN07 against oxidized low-density lipoprotein (Ox-LDL)-induced pro-inflammatory responses of human aortic smooth muscle cells [7]. Therefore, a selective PPARγ antagonist GW9662 was used to examine the involvement of PPARγ in AN07 protection on LPS-stimulated inflammatory and oxidative responses in RAW 264.7 macrophages. Results indicated that the combined treatment of GW9662 (10 μM) in LPS-stimulated RAW 264.7 cells significantly attenuated the effect of AN07 (1.0 μM) on enhancing nuclear expression of Nrf2 (Figure 2D) and down-regulating COX-2 (Figure 4). These results suggested the involvement of PPARγ in the protective effects of AN07 against LPS-induced inflammatory responses and oxidative stress in RAW 264.7 macrophages.

### 2.3. AN07 Increases Cell Viability and Decreases Apoptotic Cell Death in MG-Treated SH-SY5Y Cells

**Figure 5:**
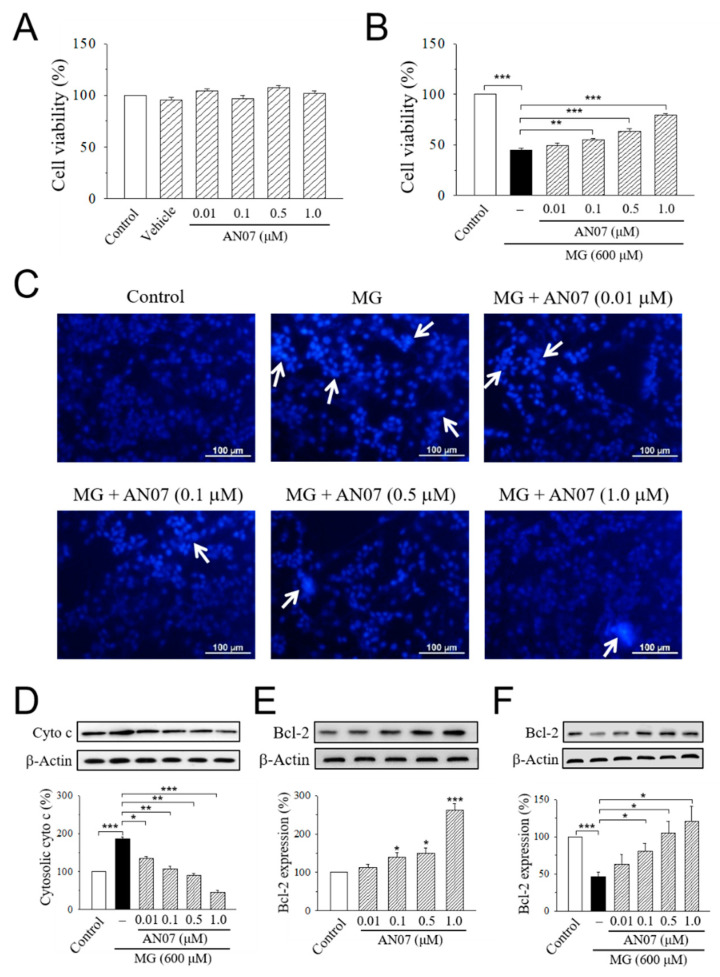
AN07 protects against methylglyoxal (MG)-induced apoptotic death in SH-SY5Y cells. (**A**,**B**) Effects of AN07 on cell viability of normal (**A**) or MG-treated (**B**) SH-SY5Y cells. SH-SY5Y cells were pre-treated with AN07 (0.01–1.0 μM) for 1 h and then exposed to 600 μM MG for another 24 h. Cell viability was determined by MTT assay. (**C**) Representative fluorescent images showing the protective effects of AN07 on apoptotic nuclear condensation. Nuclear condensation (white arrow) was determined by Hoechst 33342 staining and observed by a fluorescent microscope. Scale bar, 100 µm. (**D**) Effects of AN07 on cytosolic cytochrome c (cyto c) expression in MG-treated cells. (**E**,**F**) Effects of AN07 on B-cell lymphoma 2 (Bcl-2) expression in normal (**E**) or MG-treated (**F**) cells. Upper panels, representative immunoblots. Lower panels, densitometry analyses of the relative ratio of protein/β-actin protein, represented as percentages of the control group. Columns, mean ± S.E.M. from at least three independent experiments. * *p* < 0.05, ** *p* < 0.01, *** *p* < 0.001, compared with control or indicated groups.

We next investigate the neuroprotective effect of AN07 against MG-induced neurotoxicity in human dopaminergic SH-SY5Y cells. Results from MTT assay indicated that the exposure of SH-SY5Y cells to AN07 at 0.01–1.0 μM did not affect cell viability (Figure 5A). Treatment with MG (600 μM) significantly decreased cell viability to less than 50%, and pre-treatment with AN07 at 0.01–1.0 μM dose-dependently attenuated MG-induced cell viability loss (Figure 5B). To ascertain the cytoprotective effects of AN07 on MG-induced SH-SY5Y cell death, Hoechst 33342 staining was performed to detect the features of apoptotic cells (nuclear condensation). As shown in Figure 5C, MG induced nuclear condensation in SH-SY5Y cells (white arrows), which was reduced by the pre-treatment with AN07 at 0.01–1.0 μM. Additionally, results from Western blot analyses showed that AN07 pre-treatment at 0.01–1.0 μM decreased MG-induced cytosolic expression of cytochrome c (Figure 5D). Moreover, MG stress resulted in a significant decrease in the protein levels of anti-apoptotic protein B-cell lymphoma 2 (Bcl-2), which was markedly restored by AN07 pre-treatment (Figure 5F). AN07 also up-regulated the protein expressions of Bcl-2 in SH-SY5Y cells (Figure 5E). These results suggested that the neuroprotection conferred by AN07 pre-treatment against MG-induced stress involves the protection from apoptosis.

### 2.4. AN07 Ameliorates Neurite Damage and Inhibits ROCK2/p-LIMK1 Pathway in MG-Treated SH-SY5Y Cells

**Figure 6:**
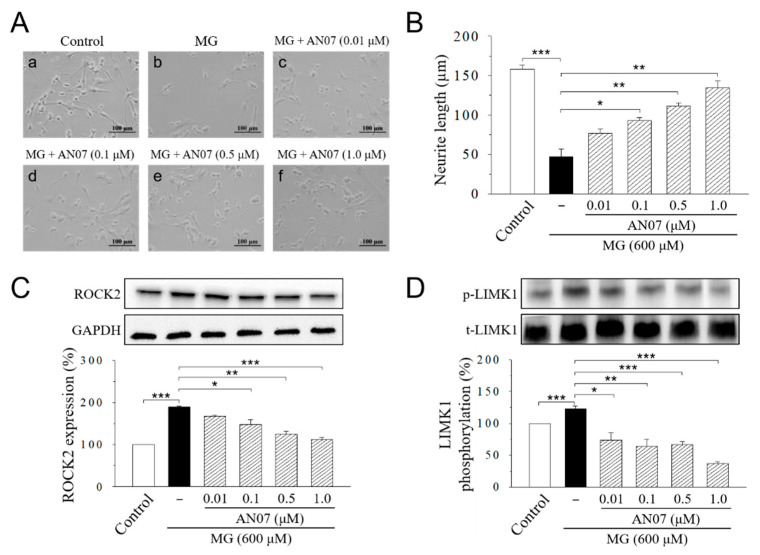
AN07 attenuates methylglyoxal (MG)-induced neurite damage in SH-SY5Y cells. (**A**,**B**) Representative images showed the protective effect of AN07 on MG-induced reduction in neurite outgrowth in SH-SY5Y cells. Cells were incubated with retinoic acid (10 μM) for five days to induce differentiation. Differentiated SH-SY5Y cells were pre-treated with AN07 (0.01–1.0 μM) for 1 h and then exposed to 600 μM MG for another 24 h. Neurites were imaged with a phase-contrast microscope (**A**), and the neurite length of cells (**B**) was calculated using the ImageJ software. Scale bar, 100 μm. Columns, mean ± S.E.M. from at least 30 cells of three independent experiments. (**C**,**D**) AN07 down-regulates Rho-associated protein kinase 2 (ROCK2) expression and LIM kinase 1 (LIMK1) phosphorylation in MG-treated SH-SY5Y cells. Upper panels, representative immunoblots. Lower panels, densitometry analyses of the relative ratio of ROCK2/glyceraldehyde-3-phosphate dehydrogenase (GAPDH) protein and phosphorylated-LIMK1/total-LIMK1 (t-LIMK1), which is represented as percentages of the control group. Columns, mean ± S.E.M. from at least three independent experiments. * *p* < 0.05, ** *p* < 0.01, *** *p* < 0.001.

We next studied the protective effects of AN07 on neurite length by morphological analyses. Retinoic acid is known with a promoting effect on nervous system development, including neuronal survival and neurite outgrowth [36]. SH-SY5Y cells are often induced to differentiate by retinoic acid to obtain neurite outgrowth and morphological changes [36,37]. The incubation of SH-SY5Y cells with serum-free defined medium in the presence of retinoic acid (10 μM) for five days induced significant neurite outgrowth to the average length of 157.8 ± 5.8 μm (Figure 6A,B), which was drastically inhibited by the MG (600 μM) treatment to 47.2 ± 9.9 μm (Figure 6A,B). Pre-treatment of AN07 at 0.01–1.0 μM attenuated MG-induced neurite retraction in a concentration-dependent manner (Figure 6A,B). Treatment of AN07 itself at 1.0 µM did not affect neurite outgrowth and neurite length in differentiated SH-SY5Y cells (Supplementary Figure S1A,B). Then, we investigated if AN07 modulates the signaling pathway for neuritogenesis in MG-treated SH-SY5Y cells. Results from Western blot analyses indicated that MG (600 μM) treatment increased Rho-associated protein kinase 2 (ROCK2) expression and the phosphorylation of downstream LIM kinase 1 (LIMK1) (Figure 6A,D). These results suggested that AN07 possesses neurite protective effects against MG-induced neurite damage in SH-SY5Y cells.

### 2.5. AN07 Upregulates the Neurotrophic Signals in Normal and MG-Treated SH-SY5Y Cells: IGF-1R, p-Akt, p-GSK3β, GLP-1R, and BDNF

**Figure 7:**
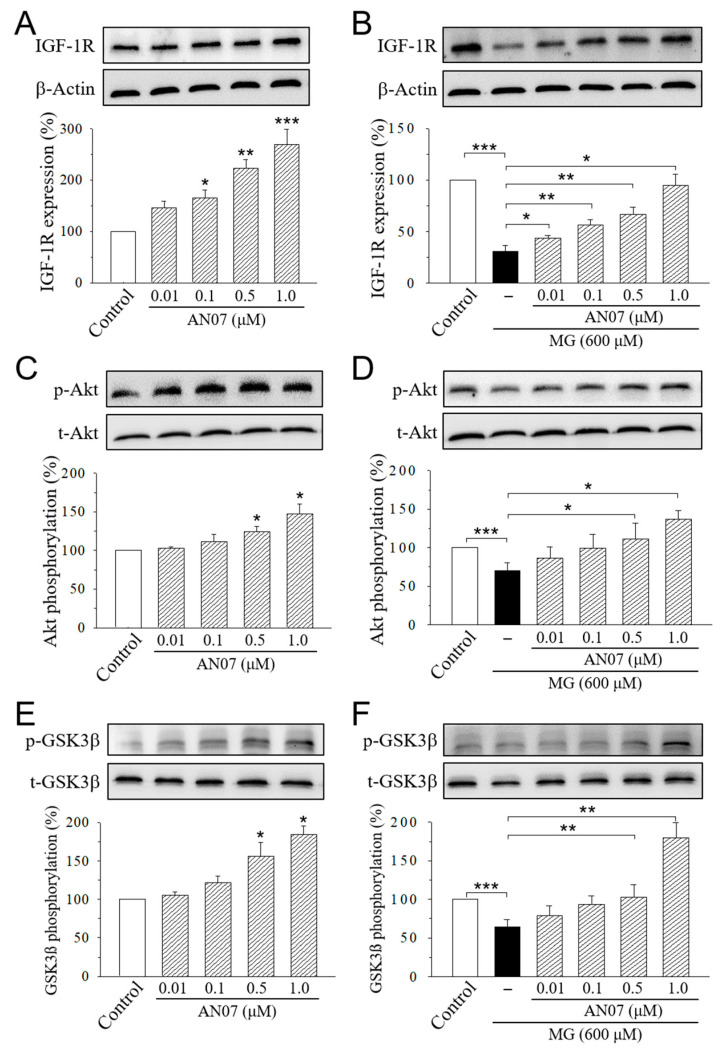
AN07 up-regulates insulin-like growth factor-1 receptor (IGF-1R) pathways in normal and methylglyoxal (MG)-treated SH-SY5Y cells. AN07 itself up-regulates IGF-1R expressions (**A**) and increases phosphorylations of Akt (**C**) and glycogen synthase kinase-3 (GSK-3β) (**E**), and AN07 pre-treatment attenuates MG-induced down-regulation of IGF-1R expression (**B**) and Akt (**D**) and GSK3ß (**F**) phosphorylations in SH-SY5Y cells. SH-SY5Y cells were pre-treated with AN07 (0.01–1.0 μM) for 1 h and then exposed to 600 μM MG for another 24 h. Upper panels, representative immunoblots. Lower panels, densitometry analyses of the relative ratio of protein/β-actin protein, or phosphorylated-protein (*p*-protein)/total-protein (*t*-protein), represented as percentages of the control group. Columns, mean ± S.E.M. from at least three independent experiments. * *p* < 0.05, ** *p* < 0.01, *** *p* < 0.001, compared with control or indicated groups.

We next examined the effects of AN07 on the pathways of neuroprotection in MG-treated SH-SY5Y cells. SH-SY5Y cells were treated with AN07 at 0.01–1.0 μM for 24 h (Figure 7A,C,E), or pre-treated with AN07 at 0.01–1.0 μM for 1 h, and then they were treated with MG (600 μM) for 24 h (Figure 7B,D,F). Significant increases in the expression of IGF-1R (Figure 7A) and the phosphorylations of downstream Akt (Figure 7C) and glycogen synthase kinase-3β (GSK3β) (Figure 7E) were found in the cells treated with AN07 at 0.01–1.0 μM. Treatment with MG (600 μM) significantly decreased the expression of IGF-1R, *p*-Akt, and *p*-GSK3β, which was attenuated by the pre-treatment with AN07 at 0.01–1.0 μM in a concentration-dependent manner (Figure 7B,D,F).

**Figure 8:**
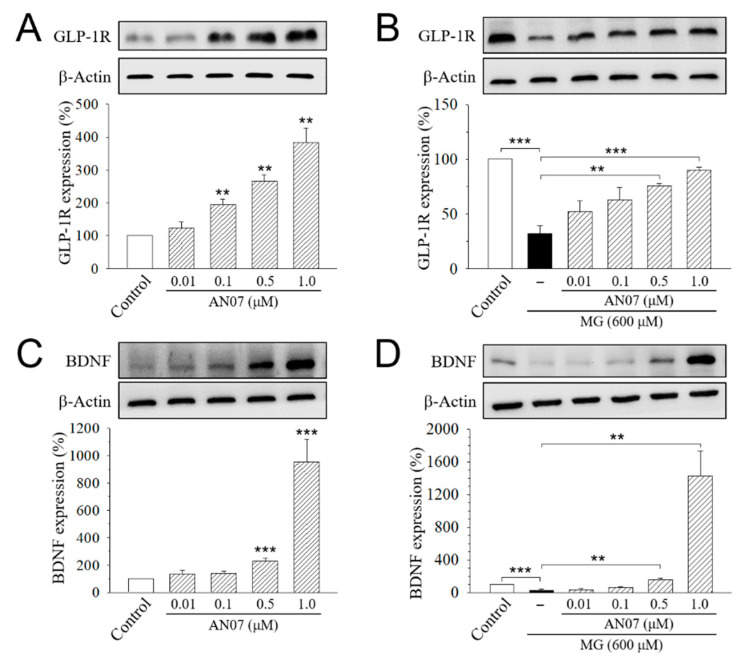
AN07 promotes the expressions of the glucagon-like peptide-1 receptor (GLP-1R) and brain-derived neurotrophic factor (BDNF) in normal and methylglyoxal (MG)-treated SH-SY5Y cells. AN07 itself up-regulates protein expressions of the GLP-1R (**A**) and BDNF (**C**), and AN07 pre-treatment attenuates MG-induced down-regulation of GLP-1R (**B**) and BDNF (**D**) in SH-SY5Y cells. SH-SY5Y cells were pre-treated with AN07 (0.01–1.0 μM) for 1 h and then exposed to 600 μM MG for another 24 h. Upper panels, representative immunoblots. Lower panels, densitometry analyses of the relative ratio of protein/β-actin protein or phosphorylated-protein (*p*-protein)/total-protein (*t*-protein), which are represented as percentages of the control group. Columns, mean ± S.E.M. from at least three independent experiments. ** *p* < 0.01, *** *p* < 0.001, compared with control or indicated groups.

We also found a significant increase in the expression of GLP-1R (Figure 8A) and BDNF (Figure 8C) in the cells treated with AN07 at 0.01–1.0 μM. Furthermore, MG (600 μM) significantly decreased the expression of GLP-1R (Figure 8B) and BDNF (Figure 8D), which was rescued by the pre-treatment with AN07 at 0.01–1.0 μM in a concentration-dependent manner. These results suggested that AN07 promotes neurotrophic signalings, including the upregulating IGF-1R pathways and increasing GLP-1R and BDNF expressions.

### 2.6. AN07 Restores the Down-Regulation of Neuroprotective Antioxidant Parkinsonism-Associated Proteins Parkin, Pink1, and DJ-1 in MG-Treated SH-SY5Y Cells

**Figure 9:**
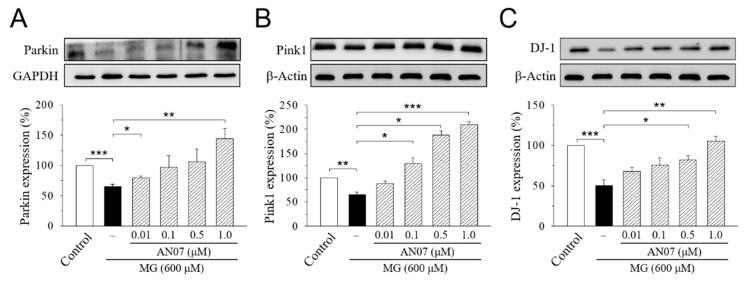
AN07 restores the down-regulation of Parkin (**A**), phosphatase and tensin homolog (*PTEN*)-induced putative kinase 1 (pink1) (**B**), and *DJ-1* (Parkinson disease protein 7, *PARK7*) (**C**) in methylglyoxal (MG)-treated SH-SY5Y cells. SH-SY5Y cells were pre-treated with AN07 (0.01–1.0 μM) for 1 h and then exposed to 600 μM MG for another 24 h. Upper panels, representative immunoblots. Lower panels, densitometry analyses of the relative ratio of protein/β-actin protein, represented as percentages of the control group. Columns, mean ± S.E.M. from at least three independent experiments. * *p* < 0.05, ** *p* < 0.01, *** *p* < 0.001.

Recent studies suggested that the three Parkinsonism-associated proteins parkin, phosphatase and tensin homolog (*PTEN*)-induced putative kinase 1 (pink1), and *DJ-1* (Parkinson disease protein 7, *PARK7*), that functionally associate with mitochondria work in parallel to protects cells from oxidative stress and confer neuroprotection in neuronal cells [38,39]. Therefore, we further investigated the cytoprotective mechanisms of AN07 against MG-induced neurotoxicity by Western blot analyses of *parkin*, *pink1*, and *DJ-1*. Results from the densitometric analyses showed a significant decrease in protein levels of parkin (Figure 9A), pink1 (Figure 9B), and *DJ-1* (Figure 9C) in MG-treated SH-SY5Y cells, which was concentration-dependently upregulated by the pre-treatment of AN07 at 0.01–1.0 μM (Figure 9A–C), suggesting that the neuroprotection conferred by AN07 pre-treatment against MG-induced neurotoxicity involves the parkin/pink1/*DJ-1* pathway. These results also implied the antioxidant potentials of AN07 against MG-induced neuronal damage.

## 3. Discussion

Chalcones are a group of aromatic enones that belong to the flavonoid family and have been widely investigated for various pharmacological activities such as anti-tumor, antioxidant, anti-inflammatory, anti-diabetic, and neuroprotective effects [2,3]. Many naturally occurring or synthetic chalcone derivatives have been reported for anti-inflammatory potentials [40,41]. Since chalcones are easy to synthesize, it is possible that naturally occurring chalcones may serve as lead compounds for the further enrichment of the structural diversity or the enhancement of its safety or efficacy for a targeted pharmacological profile. AN07, a novel chalcone derivative synthesized in our previous research, has been demonstrated to inhibit Ox-LDL-induced expression of the pro-inflammatory cytokines such as IL-1β and IL-6 in human aortic smooth muscle cells [6]. In an attempt to take advantage of chalcone-derived multifarious activities, this study was designed to investigate the dual anti-inflammatory and neuroprotective potentials of AN07. Using the model of LPS-stimulated RAW 264.7 macrophages, the present study demonstrated the anti-inflammatory activity and antioxidant potentials of AN07. With the neurotoxic model of human dopaminergic SH-SY5Y cells stressed with MG, we also revealed the neuroprotective capabilities of AN07 by mechanisms involving the increase in cell survival, neurite outgrowth, and neurotrophic signals.

When inflammation occurs, activated macrophages release a large number of different inflammatory mediators, including NO, PGE_2_, TNF-α, and IL-6, and regulatory enzymes such as iNOS and COX-2 [8]. iNOS catalyzes the production of NO, which is a free radical with diverse physiological and pathophysiological functions. Excess NO production could induce deleterious effects, including tissue damages and inflammatory responses [42]. COX-2 is an important enzyme involved in the biosynthesis of the pro-inflammatory lipid mediator PGE_2_ [43]. In the current study, LPS stimulation elevated the levels of the inflammatory mediator NO and regulatory enzymes iNOS and COX-2 in RAW 264.7 macrophages, but such effects were significantly ameliorated by AN07 pre-treatment, suggesting the prominent anti-inflammatory activities of AN07.

It has been reported that activation of the antioxidant Nrf2 pathway suppresses inflammatory responses in LPS-stimulated macrophages by blocking the transcriptions of pro-inflammatory cytokines, including IL-6 and IL-1β [44]. Increasing the expressions of HO-1 can reduce LPS-induced production of iNOS and NO in macrophages [45], suggesting the importance of HO-1 in oxidative stress and inflammation conditions. In this regard, the Nrf2/HO-1 dependent antioxidant pathway is a potential pharmacological target for the treatment of inflammatory disorders. The present results showed that AN07 attenuated ROS generation in LPS-stimulated RAW 264.7 cells, which is accompanied by reduced gp91^phox^ (NOX2) expressions. AN07 promoted the nuclear expression of Nrf2, which in turn increased the downstream antioxidant protein HO-1 and total GSH level, and thus reduced cellular ROS levels in LPS-induced RAW 264.7 cells. Moreover, in accordance with previous studies showing that the high expression of HO-1 can inhibit LPS-induced NO production [46], AN07 reduced LPS-induced NO production accompanied by increasing HO-1 expression. These results suggested, for the first time, that AN07 activates the Nrf2/HO-1 pathway and up-regulates antioxidant effectors, which may in turn reduce inflammation and oxidative stress.

PPARγ belongs to the superfamily of ligand-activated transcription factors and possesses several biological functions. Previous studies have indicated that PPARγ agonists possess in vitro and in vivo anti-inflammatory effects by suppressing nuclear factor kappa B (NF-κB) activation and inhibiting the release of pro-inflammatory cytokines [47]. The previous study in human aortic smooth muscle cells has demonstrated that AN07 is an inducer of PPARγ, and PPARγ-dependent mechanisms are involved in the inhibitory effects of AN07 against Ox-LDL-induced inflammatory responses [7]. Consistent with the previous observation, the current results indicated that the protective effects of AN07 in LPS-stimulated RAW 264.7 macrophages, including up-regulating nuclear Nrf2 expression and down-regulating COX-2 expression, were abrogated by the combined treatment of a selective PPARγ antagonist GW9662. We also found that LPS-induced IκBα phosphorylation was attenuated by AN07, suggesting the inhibition of the NF-κB pathway. Therefore, combined the results from previous and current investigations, it is suggested that AN07 exerts the antioxidant and anti-inflammatory effects, at least partially through the mechanisms involving the activating PPARγ or inhibiting NF-κB pathway.

Accumulating studies revealed the neuroprotective activities of naturally occurring or synthetic chalcone derivatives. For example, a chalcone-enriched fraction isolated from the stem bark of a Brazilian medicinal plant, *Myracrodruon urundeuva*, presents neuroprotective actions on 6-hydroxydopamine-induced neuronal cell death in rat mesencephalic cells [48], implying the beneficial effects of chalcones in neurodegenerative injuries such as Parkinson’s disease. A recent study also reported the neuroprotective effects of a synthetic halogen-containing chalcone derivative in SH-SY5Y cells by enhancing neurotrophic signal, antioxidant defense, and the anti-apoptosis pathway [49]. The present study investigated the neuroprotective effects of AN07 against MG-induced neurotoxicity in SH-SY5Y cells. AN07 up-regulated the protein expression of IGF-1R, GLP-1R, and BDNF and increased the phosphorylation of downstream Akt and GSK-3β, as well as promoted the levels of anti-apoptotic protein Bcl-2, in SH-SY5Y cells treated with or without MG, suggesting that the neuroprotective mechanisms of AN07 involve, at least, the decrease in apoptosis, the activation of IGF-1R and GLP-1R signals, and the promotion of neurotrophic BDNF expression. Interestingly, our current results from the short-term treatment (24 h) of AN07 in SH-SY5Y cells showed no significant differences in the neuronal viability and neurite outgrowth. Further studies using more extended treatment protocols or different study models are required to investigate the potentials of AN07 as a neurotrophic compound promoting neuronal survival, differentiation, or neurite outgrowth.

Previous studies indicated that in addition to the apoptotic death, MG-induced neural damage involves the failures of neurite outgrowth [22]. The Rho/ROCK pathway is an important adverse regulating pathway of neurite outgrowth [50]. The activation of ROCK leads to LIMK phosphorylation, which then inactivates cofilin by phosphorylation and induces the actin cytoskeleton rearrangement, ultimately resulting in neurite collapse [51]. Neurite collapse is one of the features of diabetic neuropathy [52]. Recent studies suggested the inhibition of the ROCK pathway as a potential neuroprotective strategy for diabetic neurodegeneration. For example, the pharmacological inhibition of RhoA and downstream ROCK pathway improved neurite outgrowth and the neuronal differentiation of mouse neural stem cells [53]. Our results from morphological analyses of neurite outgrowth demonstrated that AN07 attenuated MG-induced neurite retraction in differentiated SH-SY5Y cells. AN07 also attenuated ROCK2 expression and LIMK1 phosphorylation in MG-treated SH-SY5Y cells, suggesting that the mechanisms of AN07-mediated neuroprotection on neurite outgrowth involves, at least, the inhibition on the ROCK/LIMK pathway.

Parkinson’s disease (PD) is a common and incurable neurodegenerative disease. The connection between diabetes and PD has been a subject of attention for a long time [54] in particular because of the similar dysregulated pathways, such as mitochondrial dysfunction, endoplasmic reticulum stress, inflammation, and alterations in metabolism in the pathogenesis of both diseases [55]. Most cases of PD are sporadic and of unknown origin, but the discovery of various genes linked to familial forms of PD confirms the role of genetics in disease development [56]. Among those PD-related genes, *parkin*, *pink1*, and *DJ-1* are involved in the early-onset recessive Parkinsonism and functionally associated with mitochondria [57]. Parkin is an E3 ubiquitin ligase that is recruited to mitochondria after the loss of mitochondrial membrane polarization [58]. Pink1 is a mitochondrial serine/threonine kinase on the outer mitochondrial membrane [59]. *DJ-1* functions as a redox-responsive cytoprotective protein that plays a crucial role in antioxidant and neuroprotection in neuronal cells [60]. It has been shown that *DJ-1* works in parallel with the parkin/pink1 pathway to maintain the mitochondrial function of human neuroblastoma cells in the presence of oxidative stress [39]. *DJ-1* was reported to have novel deglycase or glyoxalase activity by which the enzymatically removing early-stage MG and glyoxal adducts from protein side-chains prevent the formation of irreversible AGEs [61,62]. The reported enzymatic activities of *DJ-1* as a glyoxalase or a deglycase would create a link between diabetes and PD, since *DJ-1* has been associated with Parkinsonism and MG detoxification [63,64]. Thus, strategies for up-regulating protein expression or augmenting the activity of parkin/pink1/*DJ-1* pathway might provide useful therapeutic approaches for neurodegenerative complications, such as diabetes and PD [65,66]. The data from the current study demonstrated, for the first time, that AN07 elicits cytoprotective effects against MG-induced neurotoxicity through the mechanisms involving the up-regulation of antioxidant Parkinsonism proteins *parkin*, *pink1*, and *DJ-1*. Further investigation would contribute to the understanding of whether AN07 could exert similar neuroprotective effects against Parkinsonian neurodegenerative disorders through the up-regulation of the parkin/pink1/*DJ-1* pathway.

It has been shown that the neuroprotective activity of antioxidants involves the direct neuroprotection against oxidative stress per se in neurons, as well as the indirect protection via the suppression of pro-inflammatory cytokines and other mediators released from glial cells [67,68]. The findings from these two cell models in the present study provide the rationale for further investigation into the potentials of AN07 on inflammation- and oxidative stress-related secondary neuronal damage or neurodegenerative disorders. Further work using the coculture of the microglial-like cells with the neuronal-like cells may help clarify the effects of AN07 on the suppression of glia-mediated inflammation.

Accumulating in vitro studies have shown that dietary flavonoids inhibit the formation of AGEs by trapping MG, the reactive dicarbonyl species precursor of AGEs [17], under physiological conditions [69,70,71,72]. Based on the observations on different flavonoids, compounds with the catechin-like A-ring structure showed the most potent effect on direct trapping of MG. Besides, the trapping ability is mainly determined by the total number and position of the hydroxyl groups. Chalcone, a precursor for the synthesis of other flavonoids in plants, is a type of open-chain flavonoid with the middle three carbon atoms not forming a closed ring. It is reasonable to hypothesize that chalcones that have the same A ring structure as catechin can trap MG and quench the deleterious effect of MG. Indeed, two chalcones, phloretin and phloridzin, have been shown for the trapping activity of MG [73]. Therefore, an interesting question would be raised: “Is there any chance for a direct trapping mechanism of AN07 on attenuating MG-induced neurotoxicity?” Considering the structure features of AN07 with only one hydroxyl group, the MG-trapping activity of AN07 may be lower than the naturally occurring chalcones. However, further investigation using in vitro study models is required to precisely determine the MG trapping ability of AN07. Moreover, the difference in the concentration range of AN07 (0.01–1.0 µM) and MG (600 µM) employed in this study also reduce the contribution of MG trapping potentials of AN07 toward its neuroprotective effects.

Taken together, the herein presented data revealed that AN07 possesses antioxidant activities against LPS-induced inflammation and elicits cytoprotective effects against MG-induced neurotoxicity. AN07 possesses significant anti-inflammatory and antioxidant potentials in LPS-stimulated RAW 264.7 macrophages by inhibiting the generation of ROS, decreasing the production of the inflammatory mediator NO and regulatory enzymes iNOS and COX-2, and up-regulating the antioxidant Nrf2/HO-1 pathways, suggesting the therapeutic potentials of AN07 for inflammatory disorders. Our results also demonstrated that, for the first time, AN07 attenuated MG-induced apoptotic death and neurite damage in SH-SY5Y cells via the up-regulation of IGF-1R, GLP-1R, and BDNF, the promotion of antioxidant Parkinsonism proteins *parkin*, *pink1*, and DJ1, and the inhibition of the ROCK/LIMK pathway, demonstrating the novel neuroprotective mechanisms of AN07 against neuronal disorders related to AGE accumulation.

## 4. Materials and Methods

### 4.1. Chemicals and Reagents

The method for the synthesis of AN07 (2-hydroxy-4′-methoxychalcone) was described in detail in the previous studies [6,7]. The purity of AN07 was higher than 95%, as determined by high-performance liquid chromatography. The chemical structure of AN07 is shown in Figure 1. LPS, MG, 3-(4,5-dimethylthiazol-2-yl)-2,5-diphenyl-tetrazolium bromide (MTT), dimethyl sulfoxide (DMSO), Hoechst 33342, 2′,7′-dichlorodihydrofluorescein diacetate (H2DCF-DA), and GW9662 were obtained from Sigma-Aldrich (St. Louis, MO, USA). Dulbecco’s modified Eagle’s medium (DMEM), fetal bovine serum (FBS), lysis buffer, and nuclear extraction kit were obtained from Thermo Fisher Scientific (Waltham, MA, USA). A mitochondria/cytosol fractionation kit was purchased from BioVision (Milpitas, CA, USA). A GSH quantitation kit was obtained from Enzo Life Sciences (Farmingdale, NY, USA). Antibodies used for immunoblotting were as follows: β-actin and iNOS from Sigma-Aldrich (St. Louis, MO, USA); Bcl-2, cytochrome c, *p*-GSK3β, Nrf2, Lamin B, HO-1, COX2, donkey anti-goat IgG-horseradish peroxidase (IgG-HRP), goat anti-mouse IgG-HRP, and goat anti-rabbit IgG-HRP from Santa Cruz Biotechnology (CA, USA); *p*-IκBα, IκBα, IGF-1R, *p*-Akt, *t*-Akt, *t*-GSK3β, ROCK2, *p*-LIMK1, *t*-LIMK1, *Parkin, pink1*, and *DJ-1* from Cell Signaling (Danvers, MA, USA); BDNF and GLP-1R from Abcam (Cambridge, UK); gp91^phox^ from BD Biosciences (San Jose, CA, USA); GAPDH from GeneTex (Irvine, CA, USA). Enhanced chemiluminescence reagent and polyvinylidene difluoride (PVDF) membrane were obtained from Millipore (Billerica, MA, USA). All materials for SDS-PAGE were obtained from Bio-Rad (Hercules, CA, USA).

### 4.2. Cell Culture and Chemical Treatment

Murine macrophage cell line RAW 264.7 and human dopaminergic cell line SH-SY5Y were obtained from the American Type Culture Collection (Rockville, MD, USA). RAW 264.7 and SH-SY5Y cells were maintained at 37 °C in a humidified incubator with 5% CO2 and 95% air and cultured in Dulbecco’s Modified Eagle Medium (DMEM) containing 10% (*v*/*v*) heat-inactivated FBS, 4 mM glutamine, 100 U/mL penicillin, 100 μg/mL streptomycin, and 0.25 μg/mL amphotericin B. The cell culture medium was replaced every 2–3 days, and confluent cells were passed every 3–5 days with trypsinization. LPS is a potent inducer of inflammatory responses, and LPS-stimulated RAW 264.7 cells have been extensively used as an in vitro model for investigating the anti-inflammatory responses and antioxidant defense mechanisms of bio-active products. Cells were treated with vehicle (0.1% DMSO) or AN07 (0.01–1.0 μM) for 24 h, or pre-treated with vehicle (0.1% DMSO) or AN07 (0.01–1.0 μM) for 1 h, and then treated with or LPS (100 ng/mL, dissolved in sterile ultrapure water (ddH_2_O)) for 24 h. SH-SY5Y cells have been commonly used as an in vitro model to investigate the pathogenesis and therapeutic strategies of MG-induced neurotoxicity [22,49]. To investigate the neuroprotective effects of AN07, cells were plated at a density of 5 × 10^5^ cells/mL medium in 6- or 96-well sterile plates in culture medium for 24 h. Cells were treated with vehicle (0.1% DMSO) or AN07 (0.01–1.0 μM) for 24 h, or pre-treated with vehicle (0.1% DMSO) or AN07 (0.01–1.0 μM) for 1 h, and then treated with MG (600 μM, dissolved in sterile ddH2O) for 24 h.

### 4.3. Cell Viability Assay

Cell viability was determined by MTT assay. Briefly, cells were plated onto 96-well plates for 24 h, and the treatments were applied. After the treatment of the cells with the indicated drug concentrations and incubation time, the culture medium was removed. Then, cells were incubated with 0.5 mg/mL MTT for 3 h at 37 °C in a humidified atmosphere of 5% CO2. Then, the medium was carefully removed, and 100 μL of DMSO for 30 min was utilized to dissolve the formazan crystals. The absorbance at 560 nm was measured in a plate reader (Thermo Fisher Scientific, USA).

### 4.4. Measurement of Reactive Oxygen Species (ROS)

Intracellular ROS production was measured using 2′,7′-dichlorodihydrofluorescein diacetate (H2DCF-DA), which readily diffuses inside cells and is cleaved by intracellular esterases, leading to the generation of the fluorescent compound 2′,7′-dichlorofluorescein (DCF), which indicates intracellular ROS levels. Briefly, in the final 30 min of incubation time with each treatment, cells were loaded with 10 μM H2DCF-DA for 30 min at 37 °C to allow cellular incorporation. Then, cells were washed with phosphate-buffered saline (PBS). One hundred thousand cells were analyzed at an excitation of 495 nm and emission of 520 nm using the Coulter CyFlow Cytometer (Partec, Germany).

### 4.5. Western Blot Analyses

Cellular and nuclear protein extracts were collected by using lysis buffer or a commercial kit, respectively (Thermo Fisher Scientific, USA). Cytosolic protein extracts were prepared by using the commercial mitochondria/cytosol fractionation kit (BioVision, CA, USA). Cell lysates were centrifuged at 15,000× *g* for 30 min at 4 °C, and the supernatant containing proteins was collected. Protein concentration was determined by using the Bio-Rad protein assay kit. Equal amounts of protein were separated on the polyacrylamide gel and transferred to PVDF membranes. Membranes were incubated with TBST (50 mM Tris-HCl, pH 7.6, 150 mM NaCl, 0.1% Tween 20) containing 5% non-fat milk for 1 h at room temperature to block the non-specific binding, and then they were incubated with the specific primary antibodies at 4 °C for overnight. Then, membranes were incubated with one of the following secondary antibodies: goat anti-rabbit IgG-HRP, goat anti-mouse IgG-HRP, or donkey anti-goat IgG-HRP for 1 h at room temperature. Protein bands were visualized with the enhanced chemiluminescence reagent. Bands in the immunoblots were quantified using the ImageJ software (National Institutes of Health, Bethesda, MD, USA).

### 4.6. Measurement of Glutathione (GSH) Level

A GSH detection kit was purchased from Enzo Life Sciences (Farmingdale, NY, USA). The principle of the GSH quantitation kit is based on the production of yellow-colored 5-thio-2-nitrobenzoic acid (TNB) from GSH and 5,5′-dithiobis-(2-nitrobenzoic acid) (DTNB). Absorbance was measured by measurement by ELISA reader (Thermo Fisher Scientific, Waltham, MA, USA) at 405 nm for 10 min at 1-min intervals. The total amount of GSH was determined using a calibration curve and normalized to the protein concentration.

### 4.7. Measurement of Nitrite Production

Nitric oxide (NO) release was indicated as nitrite generation, which was measured by the Griess reagent (1% sulfanilamide and 0.1% *N*-1-naphthylethylenediamide in 5% phosphoric acid). First, 150 μL of the medium from each group were incubated with Griess reagent at room temperature for 10 min avoiding light exposure, and then the absorbance was measured at 540 nm (OD540). The concentration was calculated from a standard reference curve of sodium nitrite.

### 4.8. Hoechst 33342 Staining Assay

The fluorescent dye Hoechst 33342 was used to detect DNA condensation, nuclear fragmentation, and characteristic features of apoptotic cells. SH-SY5Y cells were seeded in 6-well plates at a density of 5 × 105 for 24 h. After the treatment of the cells with the indicated drug concentrations and incubation time, the culture medium was removed. Then, cells were washed with PBS and fixed with 4% paraformaldehyde for 15 min. The fixed cells were washed with PBS and stained with 5 μg/mL Hoechst 33342 for 15 min. Following incubation, the cells were washed with PBS, and the cells were observed under a fluorescence microscope (Nikon, Japan).

### 4.9. Measurement of Neurite Length

To measure the neurite length, SH-SY5Y cells were differentiated by the serum-free defined medium in the presence of retinoic acid (10 μM) for five days before drug treatment. Cells were imaged with a phase-contrast microscope (Nikon, Japan), and the neurite length of cells was analyzed using the ImageJ software (National Institutes of Health, Bethesda, MD, USA). Processes with a length equal to two or more cell body diameters were scored as neurites. Neurite outgrowth was analyzed by measuring the length of neurite for all identified positive neurite-bearing cells, and the mean of the neurite length per cell was then calculated.

### 4.10. Statistical Analyses

Statistical analysis was performed using InStat version 4.0 (GraphPad Software, San Diego, CA, USA). Data were expressed as means ± S.E.M. Statistical significance was analyzed using one-way ANOVA followed by Dunnett’s post-hoc test for all-pair comparisons. A value of *p* < 0.05 was considered statistically significant.

## Supplementary Materials

The following are available online, Figure S1: AN07 itself does not affect the neurite outgrowth of SH-SY5Y cells.
