# Supplementary material for: The Antioxidant, Anti-Inflammatory, and Neuroprotective Properties of the Synthetic Chalcone Derivative AN07
Source: Molecules. 2020 Jun 24;25(12):2907. doi: 10.3390/molecules25122907 (PMC7355731; doi:10.3390/molecules25122907)
Supplement: Supplementary file 1 [file molecules-25-02907-s001.pdf]

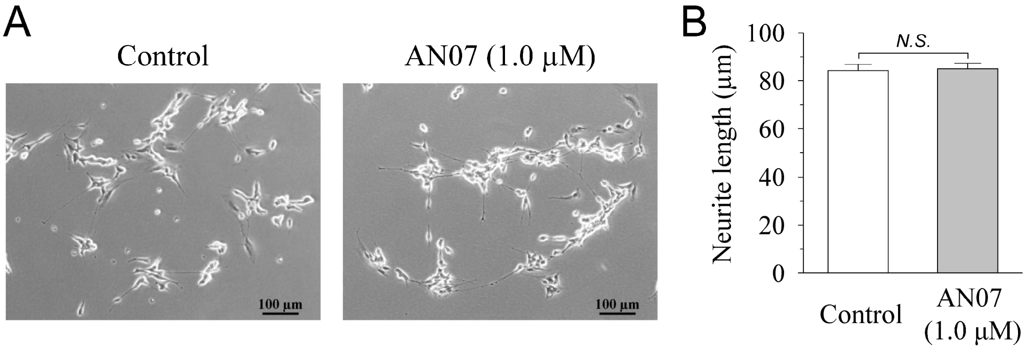

Supplementary Figure S1. AN07 itself does not affect the neurite outgrowth of SH-SY5Y cells. Cells were incubated with retinoic acid (10 μM) for five days to induce differentiation. Differentiated SH-SY5Y cells were treated with AN07 (1.0 μM) for another day. **(A)** Representative phase-contrast images showing the neurite outgrowth of SH-SY5Y cells. **(B)** Quantitative analyses of the neurite length using the ImageJ software. Scale bar, 100 μm. Columns, mean ± S.E.M. from at least 30 cells of three independent experiments. N.S. no significance.
